# S20. PARAHIPPOCAMPAL THICKNESS PREDICTS TREATMENT IMPROVEMENT IN EARLY AND CHRONIC SCHIZOPHRENIA

**DOI:** 10.1093/schbul/sby018.807

**Published:** 2018-04-01

**Authors:** Philipp Homan, Miklos Argyelan, Delbert Robinson, Todd Lencz, Anil Malhotra

**Affiliations:** 1 Feinstein Institute for Medical Research; 2 Zucker Hillside Hospital, Feinstein Institute for Medical Research

## Abstract

**Background:**

Despite recent advances, there is still a major need for prediction of treatment success in schizophrenia. Cortical thickness measures are relatively easy to obtain and may provide biomarker candidates. Here, we tested a set of candidate brain regions as predictors of treatment response in first episode schizophrenia and in two independent schizophrenia cohorts. Regions included the precuneus, inferior parietal gyrus, superior temporal gyrus, parahippocampal gyrus, anterior cingulate, inferior frontal gyrus, insula, lateral and medial orbitofrontal cortex, and occipital cortex.

**Methods:**

In the discovery cohort, we used the whole sample of patients to estimate individual response slopes using Empirical Bayes, 36 of which had cortical thickness measurements at baseline. Patients were scanned aprior to treatment with either risperidone or aripiprazole. Symptoms were assessed with the Brief Psychiatric Rating Scale at baseline and over the course of up to 52 weeks. Cortical thickness in regions of interest were examined via magnetic resonance imaging and used as predictors of individual treatment response, defined as individual response slope.

**Results:**

Parahippocampal thickness at baseline predicted the individual response to treatment (P < 0.05, Bonferroni-corrected). This was replicated in two independent schizophrenia cohorts including a recent onset cohort (N = 33) and a sample of chronic schizophrenia patients (N = 52), respectively. The overall effect was quantified with an internal meta-analysis (β = 0.4, 95% CI [0.24; 0.56]; z = 4.84, P < 0.001).

**Discussion:**

Parahippocampal thickness may be a promising marker of treatment success both at the early and the chronic stage of schizophrenia.

